# Nitro-Containing Self-Immolative Systems for Biological Applications

**DOI:** 10.3390/ph15111404

**Published:** 2022-11-14

**Authors:** Cédric Spitz, Nicolas Primas, Thierry Terme, Patrice Vanelle

**Affiliations:** 1 Aix Marseille University, CNRS, ICR UMR CNRS 7273, Equipe Pharmaco-Chimie Radicalaire, Faculté de Pharmacie, 27 Boulevard Jean Moulin—CS 30064, CEDEX 05, 13385 Marseille, France; 2 Service Central de la Qualité et de l’Information Pharmaceutiques, Hôpital de la Conception, AP-HM, 13005 Marseille, France

**Keywords:** self-immolation, nitro, prodrug, probe

## Abstract

Since its introduction in 1981, the chemistry of self-immolative systems has received increasing attention in different application areas, such as analytical chemistry, medicinal chemistry, and materials science. This strategy is based on a stimulation that triggers a cascade of disassembling reactions leading to the release of smaller molecules. The particular reactivity of the nitro group, due to its powerful electron-withdrawing nature, has been exploited in the field of self-immolative chemistry. In this context, the present review describes the major role of the nitro group in self-immolative processes depending on its position.

## 1. Introduction

**Scheme 1:**
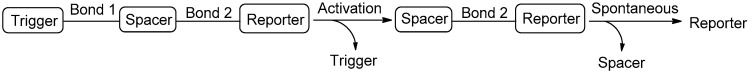
Schematic representation of a self-immolative compound.

In 1981, Katzenellenbogen et al. [1] introduced the concept of a “self-immolative connector” to overcome some problems in prodrug design. Indeed, in some cases, a bipartate prodrug A-B containing a specifier (trigger) A and a drug (reporter) B cannot be used because of the instability of the bond linking A to B, or, on the contrary the prodrug A-B is too stable or hindered to allow hydrolysis of the A-B bond. Therefore, a tripartate prodrug in which the specifier A and drug B are linked by a connector (spacer) group was considered (Scheme 1). After an appropriate stimulus, the bond between the trigger A and the spacer is broken and the remaining bond connecting the linker to the drug B must spontaneously hydrolyze to release the active drug (reporter) B. Studies on self-immolative connectors have grown significantly over the past few decades, and these degradable linkers, also named self-immolative spacers or self-immolative linkers in the literature, have found many applications in various fields, such as prodrugs, probes, imaging and materials science.

The nitro group (NO_2_) is a functional group composed of one nitrogen and two oxygens. Since the nitrogen atom bears a positive charge, the nitro group has typical reactivity due to its very strong electron-withdrawing nature. This particular reactivity has been widely used in the field of self-immolative compounds, and the nitro group can be found on the trigger, on the linker or on the reporter part. Thus, the aim of this review is to highlight the active and different roles of the nitro group in the self-immolative process depending on its position.

## 2. NO_2_ Group on the Trigger

In the first part of the review, the stimulus to initiate self-immolation directly involves a reaction with the nitro group.

### 2.1. Chemical Reduction of the Nitro Group

**Scheme 2:**
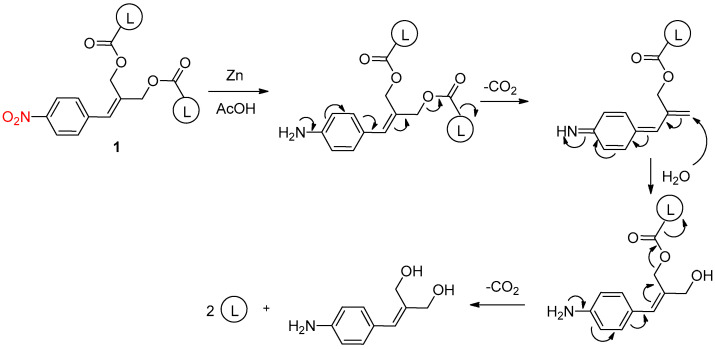
Structure of double-release compound **1** and proposed mechanism of liberation of two leaving groups L after chemical activation of **1**.

Chemical reduction of the nitro group can trigger the self-immolation cascade. For example, De Groot et al. [2] proved the concept of “cascade-release dendrimers” to liberate multiple end groups using nitro reduction as chemical activation. Dendrimers are highly ordered, treelike branched molecules. Their use in drug delivery has received much attention because biologically active substances can be attached to the ends of dendritic structures and then released by biological or chemical methods. In this approach, mild reducing conditions (Zn, acetic acid) allowed the chemical reduction of the nitro group of dendrimer **1** to the corresponding amine, followed by a self-elimination cascade to liberate two leaving groups L (Scheme 2). 

**Scheme 3:**
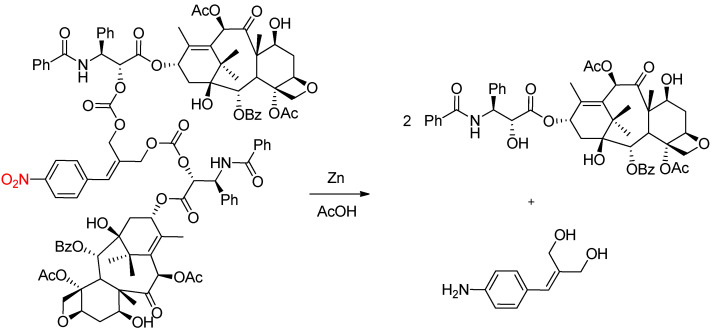
Elimination of two paclitaxel molecules in a single triggering nitro reduction of compound **2**.

This method was applied to dendron **2** to demonstrate the generality of the concept with the release of two molecules of the anticancer drug paclitaxel (Scheme 3). 

**Scheme 4:**
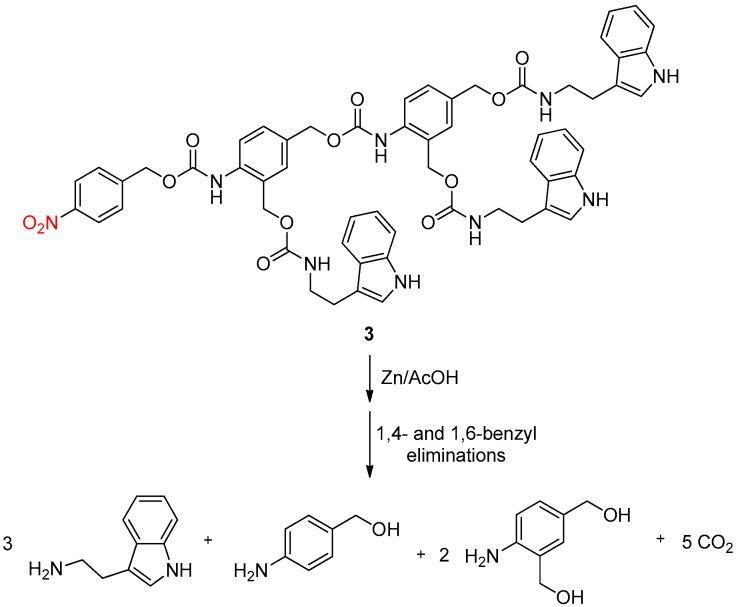
Release of tryptamine from **3** after reduction with Zn/AcOH.

Nevertheless, dendrimers have some drawbacks: the limited space available in the outer shell of the dendrimer reduces the number of convenient drug molecules. To deal with this problem, Warnecke et al. [3] described linear self-eliminating (LSE) systems that allowed the more effective release of reporter molecules after chemical activation. A linear system based on the well-known self-eliminating *p*-aminobenzyloxycarbonyl linker was envisaged. Thus, a model compound **3** with a *p*-nitrobenzyloxycarbonyl moeity and tryptamine as a reporter was synthesized. After treating **3** with zinc powder in the presence of acetic acid in acetonitrile, nitro reduction followed by 1,4- and 1,6-elimination reactions allowed the effective release of three tryptamine molecules (Scheme 4).

Here, we have shown that the chemical reduction of a nitro moiety can trigger self-immolative processes to release biologically active molecules. To apply this strategy in a biological manner, the use of nitroreductase enzymes to reduce nitro-containing compounds and then achieve self-immolation could be of great interest.

### 2.2. Enzymatic Reduction of the Nitro Group

**Scheme 5:**
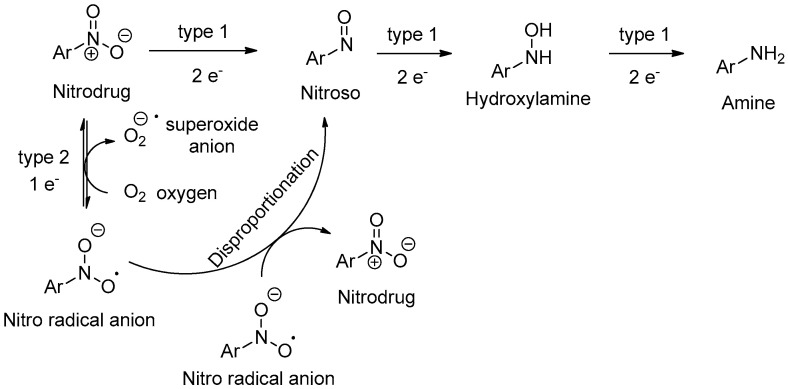
Reduction mechanisms of nitroaromatic compound by type 1 and 2 nitroreductases.

Nitroreductases, not present in humans but in bacteria or parasites, are enzymes capable of catalyzing the reduction of nitro groups. Two types of nitroreductases have been characterized: an essential mitochondrial type 1 nitroreductase catalyzing two-electron reductions and a cytosolic type 2 nitroreductase catalyzing monoelectronic reductions [4] (Scheme 5).

The toxicity of oncology drugs to non-cancer cells is the major drawback of cancer treatment. Indeed, many chemotherapy drugs lack tumor specificity and are often toxic to other tissues. Antibody-directed enzyme prodrug therapy (ADEPT) is a cancer treatment technique that consists in generating cytotoxic molecules specifically in tumors. It involves the use of a tumor-specific antibody that is linked to an enzyme and injected intravenously. Discrimination between tumor and normal tissue implies a high concentration of the antibody–enzyme conjugate in the tumor cells. Subsequently, a prodrug, administrated into the blood circulation, is converted into a cytotoxic molecule by the enzyme, selectively within the tumor.

**Scheme 6:**
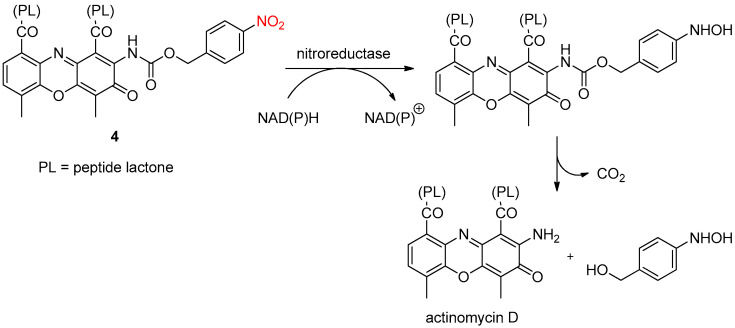
The formation of actinomycin D from its 4-nitrobenzyloxycarbonyl derivative **4** by reduction of the nitro group by the nitroreductase enzyme, followed by self-immolation of the resulting 4-(hydroxyamino)benzyloxycarbonyl derivative.

Knox et al. [5] synthesized prodrug candidates for the ADEPT strategy, which were all 4-nitrobenzyloxycarbonyl derivatives. After nitro reduction by a nitroreductase enzyme, the 4-(hydroxyamino)benzyloxycarbonyl group can generate the drug through self-immolation (Scheme 6). The authors showed that at least a 100-fold higher dose of actinomycin D was required to produce cytotoxicity equivalent to that of prodrug **4**. Moreover, prodrug **4** was 100× less toxic than actinomycin D in vivo.

Gene-directed enzyme prodrug therapy (GDEPT), using a nitroreductase, has also been the subject of considerable study to develop a more selective cancer treatment. Prodrugs activated by nitroreductase are mainly 2,4-dinitrobenzamides and 4-nitrobenzylcarbamates. Hay et al. studied the influence of substituents and leaving group effects on the fragmentation of 4-nitrobenzylcarbamates. They found that electron-donating substituents on the benzyl ring and/or α-methyl substitution in the benzylic position accelerated the fragmentation of the obtained 4-hydroxylamine derivative after enzymatic reduction [6]. On the contrary, they only observed a small influence of the leaving group on 4-hydroxylamine fragmentation [7]. 

**Figure 1:**
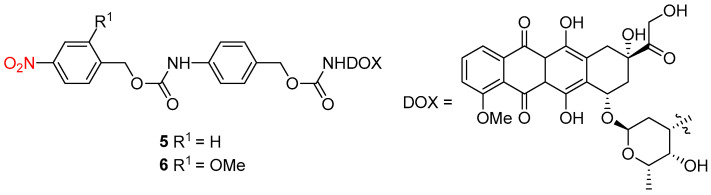
Two nitroarylcarbamate prodrugs **5** and **6** of doxorubicin.

Then, the same authors synthesized a series of nitrobenzyl carbamate prodrugs of doxorubicin [8]. Two nitrobenzylcarbamates **5** and **6** linked to doxorubicin were selective for nitroreductase across the cell line panel (Figure 1). Thus, they were selected for in vivo evaluation and were significantly less toxic than doxorubicin but were not active in vivo against nitroreductase-expressing cells in tumors, indicating that further optimization of the pharmacokinetic and pharmacodynamic parameters is required.

**Scheme 7:**
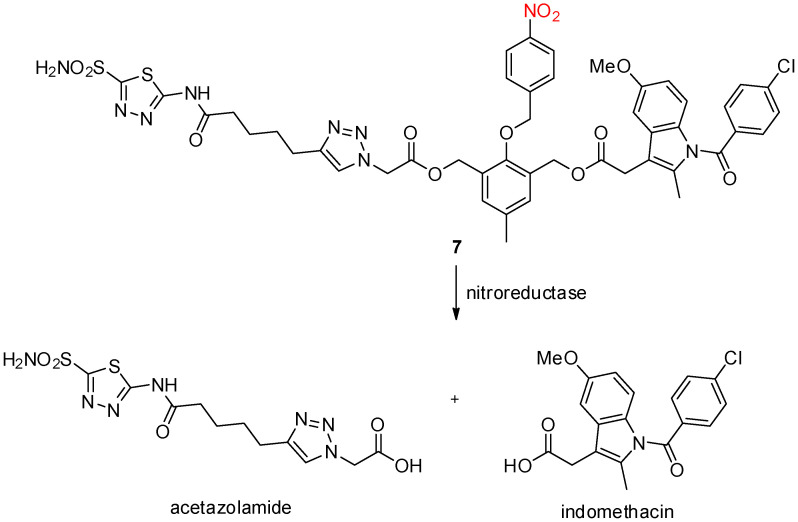
Liberation of two pharmacophores, acetazolamide and indomethacin, from prodrug **7** after nitroreductase activation.

Very recently, to attenuate cardiac inflammation, Gou et al. [9] developed a hypoxia-activated prodrug **7** consisting of a cyclooxygenase-2 inhibitor (COX-2) and a carbonic anhydrase (CA) inhibitor linked by a nitro aromatic moiety. Indeed, because cardiac inflammation is usually accompanied by hypoxia, and nitroreductase is overexpressed under hypoxia, the nitro group of prodrug **7** can be reduced by nitroreductase to an amino group. Then, intramolecular electron transfer promoted the release of two pharmacophores: indomethacin, a COX-2 inhibitor, and acetazolamide, a CA inhibitor (Scheme 7). 

In addition, prodrug **7** was stable under normoxia. Thus, it not only improved the bioavailability but also significantly reduced the side effects due to the specific release of the pharmacophores at the hypoxic site.

### 2.3. Light Activation

Previously described stimuli (chemical or biological) allowed only limited control over the time and location of delivery. To overcome this problem, the self-immolation cascade can be triggered by light activation. Indeed, the drug to deliver can be attached to a light-sensitive carrier. Then, light can be modulated (intensity, wavelength, irradiation duration) with high temporal and spatial precision in order to remotely deliver the payload. 

**Scheme 8:**
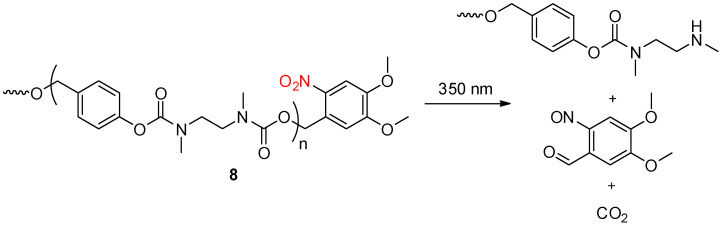
Complete degradation of photocleavable polymer **8** into small molecules after UV irradiation.

Almutairi et al. [10] developed a polymer **8** containing a *o*-nitrobenzyl alcohol (ONB) photocleavable group. After UV irradiation at 350 nm, this polymer was completely degraded into small molecules (Scheme 8).

**Scheme 9:**
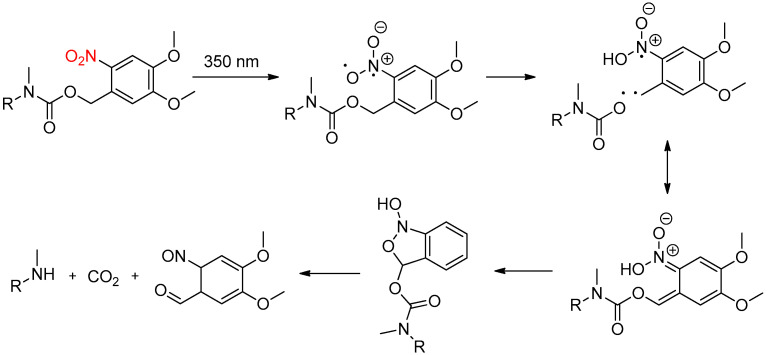
Reduction mechanism of *o*-nitrobenzyl derivatives using light.

The mechanism of the light-mediated reduction of *o*-nitrobenzyl derivatives was described by Romano et al. [11] (Scheme 9).

To exploit this property, the Nile Red (NR) dye, used as a drug model, was encapsulated using the previously synthesized polymer **8**. After UV irradiation at 350 nm, the polymeric nanoparticles released the NR payload after complete degradation.

**Scheme 10:**
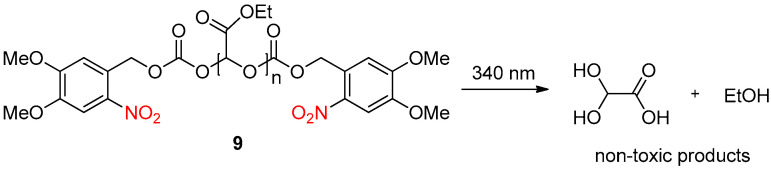
Depolymerization of polyglyoxylate **9** into non-toxic products upon UV irradiation.

Gillies et al. [12] showed that polyglyoxylates can serve as a new class of triggerable self-immolative polymers. Indeed, polymerization of readily accessible ethyl glyoxylate, followed by end-capping with a photocleavable 6-nitroveratryl carbonate (NVOC), allowed the synthesis of a photo-responsive polymer **9**. The NVOC group is well known to be cleanly cleaved under neutral UV light conditions (340 nm) and poly(ethylglyoxylate) depolymerization only provided benign products such as glyoxylic acid hydrate and ethanol (Scheme 10). Thus, they could be used for a wide range of biomedical applications, such as biomedical sutures, tissue engineering scaffolds and toxic-free drug delivery vehicles.

Furthermore, these new polyglyoxylate polymers are very interesting because the monomers can be furnished not only from petroleum-based resources but also from renewable sources.

**Scheme 11:**
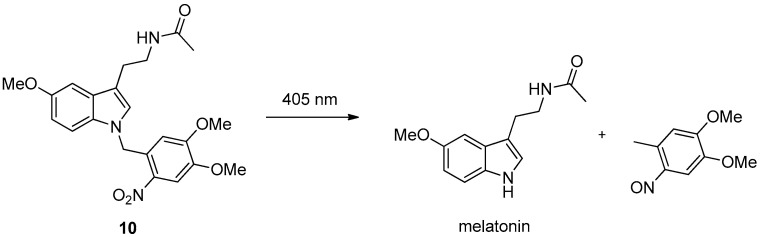
Deprotection of the ONB photocleavable group of **10** to release melatonin.

Melatonin (MT) is a hormone involved in many physiological functions, including the regulation of biological rhythms, and its secretion mainly occurs at night. However, studies on the activation of the MT1 and MT2 receptors with high spatiotemporal resolution are lacking. Very recently, Llebaria et al. [13] developed the first family of photocleavable ligands for melatonin receptors. A family of light-activable caged compounds containing a ONB photocleavable group was synthesized. Compound **10** (Scheme 11) showed the best uncaging efficiency and the most interesting properties, including chemical stability, high solubility in aqueous media and a more than 100-fold difference in affinity before and after photolysis. To trigger this photolytic process, the wavelengths generally range from 320 to 400 nm, but, here, the authors decided to use a laser with a 405 nm wavelength to avoid the toxicity and cell damage effects associated with UV light.

**Scheme 12:**
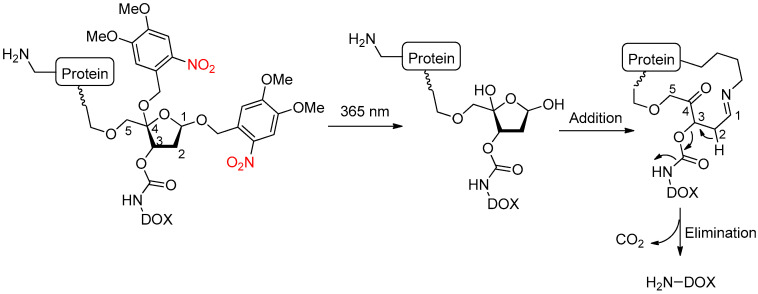
Mechanism of PC4AP-based drug delivery system.

Zhou et al. [14] designed a light-responsive, self-immolative linker for controlled drug release (Scheme 12). A photocaged C4′-oxidized abasic site (PC4AP) was used as the light-responsive, self-immolative spacer. An advantage of this linker is the ability to load an amine- or hydroxyl-bearing drug onto the C3–OH via a carbamate or carbonate bond, and the C5–OH of the PC4AP linker can be used to attach a protein, carrier peptide or antibody. The authors applied this strategy with the anticancer drug doxorubicin. Irradiation at 365 nm removed the photolabile ortho-nitrobenzyl group. Then, the N-terminal amine on the carrier peptide or protein allowed an addition–elimination cascade to finally release the drug doxorubicin.

**Scheme 13:**
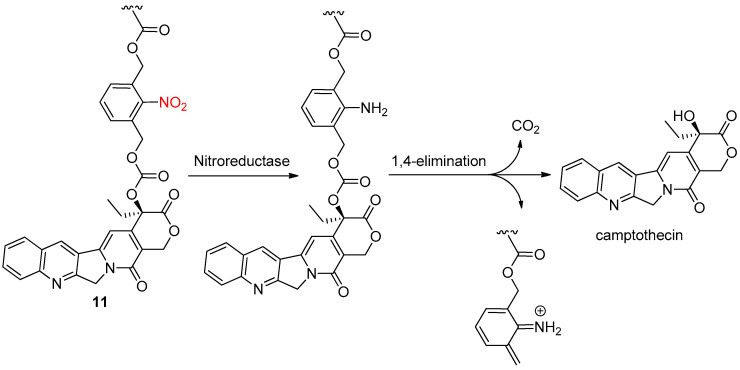
Camptothecin release from polymer **11** after near-infrared and nitroreductase activation.

Hypoxia-activated prodrugs have shown promising results in cancer therapy but the heterogeneous distribution of hypoxic areas has weakened their therapeutic activity because oxygen concentrations can remain relatively high near blood vessels. The combination of hypoxia-responsive systems and photodynamic therapy could be a promising method compared to conventional therapy. Moreover, some problems remain: many methods work upon UV irradiation with low tissue penetration ability and, in most cases, drugs are encapsulated into nanoparticles, which may imply uncontrollable drug release before reaching the targeting site. To overcome these limitations, Ge et al. [15] prepared a novel block copolymer polydrug **11** containing ortho-nitrobenzyl-linked camptothecin methacrylate to then encapsulate an indocyanine green photosensitizer with absorbance in the near-infrared range to provide deeper tissue penetration. After intravenous injection, the nanoparticles can accumulate into tumors. Upon exposure to near-infrared light, the indocyanine green inside the nanoparticles produced reactive oxygen species. Simultaneously, nitro groups were reduced to amine groups by nitroreductase assistance and self-immolative cleavage by 1,4-elimination released the therapeutic drug camptothecin (Scheme 13).

The combination of photodynamic therapy and hypoxia-activable prodrugs overcomes the limitations of each therapy alone, with a remarkable improvement in the efficacy of tumor growth suppression.

**Scheme 14:**
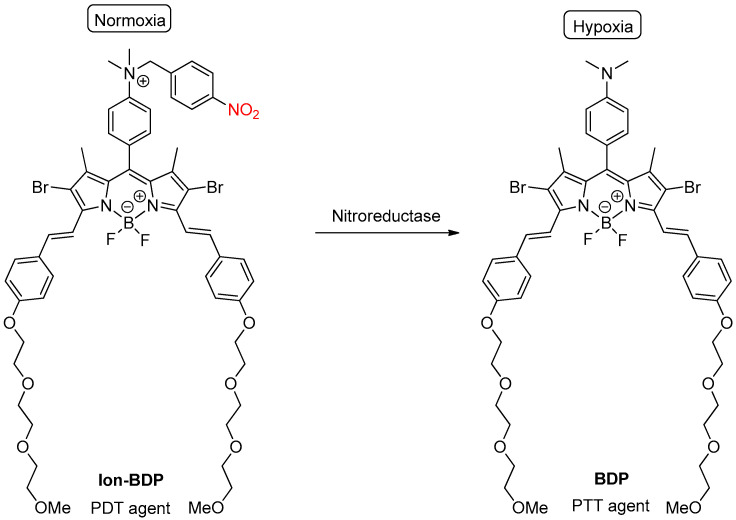
Switchable phototherapeutic strategy.

Recently, Liu et al. [16] designed a smart photosensitizing agent, Ion-BDP, which can generate reactive oxygen species upon illumination under normoxic conditions to eliminate external aerobic tumor cells. Moreover, under hypoxic conditions, the 4-nitrobenzyl group of the photosensitizer can be reduced by nitroreductase, and subsequent self-immolation allowed the formation of BDP, which is a highly effective photothermal agent (Scheme 14). 

The synergistic antitumoral effects of this photosensitizer may provide an alternative to overcome the therapeutic difficulty arising from heterogeneous oxygen distributions in solid tumors.

**Scheme 15:**
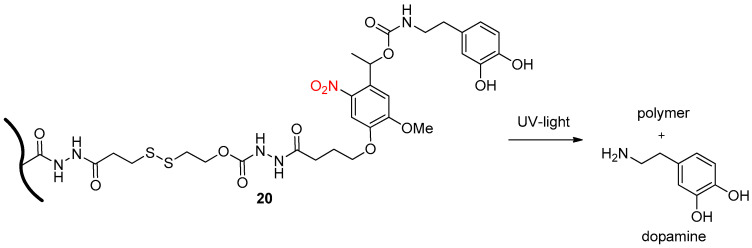
Release of dopamine after UV activation of the prodrug **20**.

Ossipov et al. [17] developed a light-activable prodrug **20** based on hyaluronan hydrogels with a photo-labile *ortho*-nitrobenzyl linker. Immobilization of a model dopamine drug into hyaluronic acid biomaterials via the photo-cleavable nitrobenzyl spacer allowed the release of dopamine upon exposure to UV light (Scheme 15).

### 2.4. Glutathione Activation

The mitochondrion is a crucial organelle present in most eukaryotic organisms and plays a fundamental role in ATP production. Mitochondria are also involved in many other tasks, such as reactive oxygen species-induced apoptosis. Thus, to protect cells from oxidative stress, mitochondrial glutathione (GSH) plays an essential role. Abnormal levels of GSH have been correlated with various diseases. Therefore, it is very interesting to develop a mitochondrial GSH probe to measure the level of mitochondrial GSH in cells.

**Scheme 16:**
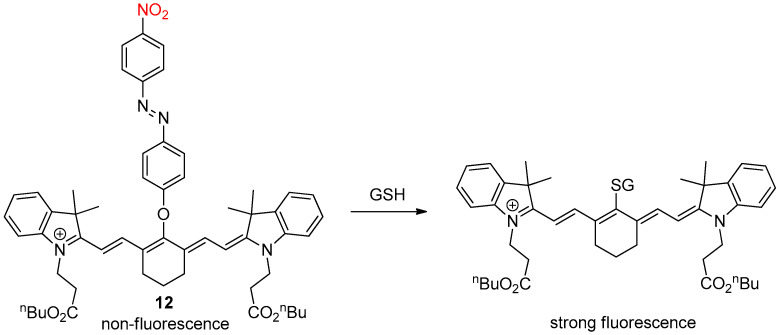
Replacement of the labile nitroazo group of compound **12** by the addition of GSH induced strong fluorescence.

Kim et al. [18] developed a heptamethine-azo conjugate as a near-infrared fluorescent probe to detect mitochondrial GSH. The initial probe **12** was nonfluorescent and contained a labile nitroazo group that can be replaced by the 1,6-conjugate addition of GSH. Subsequent elimination of the nitroazo group produced strong fluorescence (Scheme 16). Furthermore, the probe exhibited a selective fluorescence response toward GSH over the thiol-containing amino acids, cysteine and homocysteine. 

**Scheme 17:**
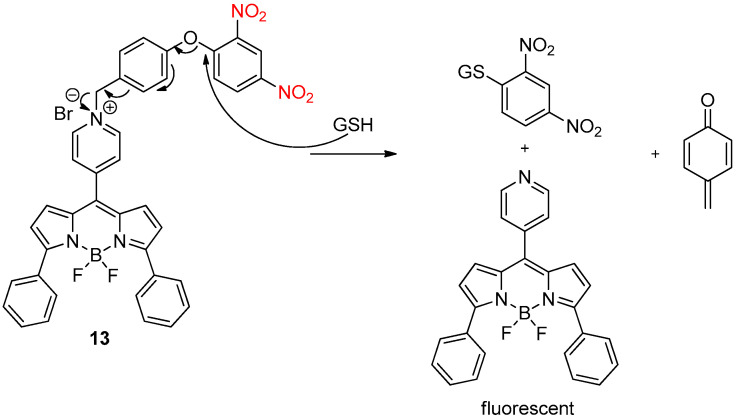
Plausible mechanism for the selective reaction of probe **13** to GSH.

Zhao et al. [19] also described a fluorescent probe **13** for the selective detection of GSH. This probe bears a *para*-dinitrophenoxybenzylpyridinium moiety in the meso position of a BODIPY dye. In the presence of GSH, the dinitrophenylether was cleaved to give a *para*-hydroxybenzyl group that can self-immolate by an intramolecular 1,4-elimination reaction to give the fluorescent BODIPY dye (Scheme 17).

**Scheme 18:**
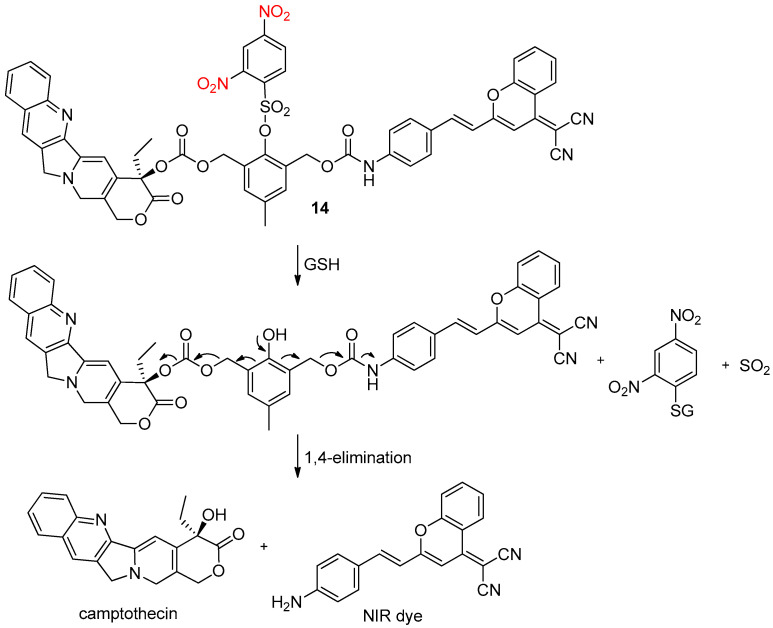
Mechanism of camptothecin and NIR dye release from the dual self-immolative system **14** after GSH activation followed by linker self-immolation.

Wu et al. [20] reported a dual self-immolative system **14** capable of delivering a drug as well as a near-infrared (NIR) fluorophore. Indeed, anticancer drug camptothecin was linked with a dicyanomethylene-*4H*-benzopyran NIR fluorophore by a nitro-containing cleavable spacer (Scheme 18). 

The presence of GSH triggered a cascade of elimination reactions that not only liberated the active anticancer drug camptothecin but also an NIR fluorophore to generate strong NIR fluorescence. This strategy has been successfully applied for the in vivo and in situ tracking of drug release and cancer treatment in a mouse model.

**Scheme 19:**
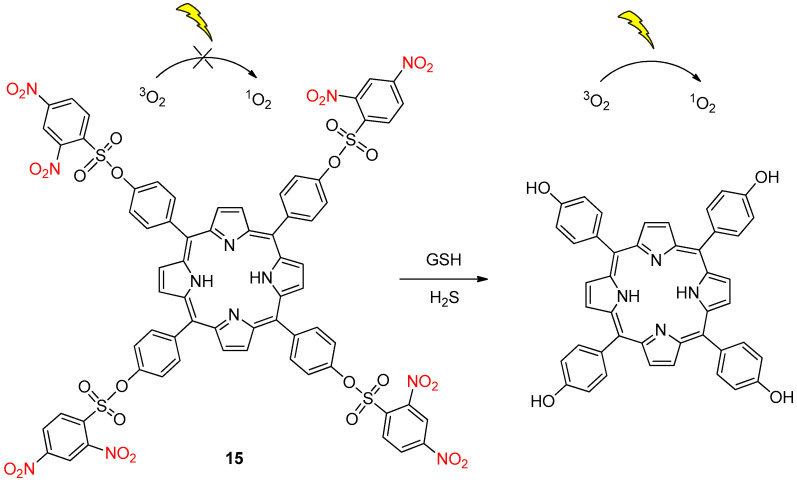
The activation mechanism of photosensitizer **15**.

Very recently, Chen et al. [21] developed the first GSH and hydrogen sulfide dual-responsive photosensitizer **15** (Scheme 19). Tetra-substituted porphyrins were chosen to minimize the side effects of **15** by quenching its photoactive activity. Then, activation of designed photosensitizer **15** by GSH and hydrogen sulfide, overexpressed in cancer cells, allowed the deprotection of the 2,4-dinitrobenzenesulfonyl moieties to restore phototoxicity to the cancer cells. Thus, phototoxicity to normal cells was reduced by the quenching effect.

## 3. NO_2_ Group on the Linker

In this second part, the nitro group is directly borne by the self-immolative linker of the molecule.

The ADEPT strategy, described previously in this review, was based on nitro reduction by a nitroreductase. Here, the involved enzymes are not nitroreductases. Thus, the nitro group present on the prodrug linker was not reduced. Indeed, the ADEPT strategy can also be used with *β*-D-glucuronidase because tumors have a high extracellular concentration of this enzyme.

**Scheme 20:**
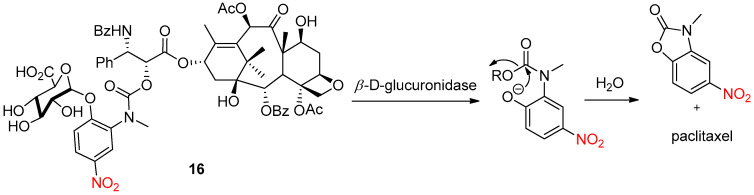
Release of paclitaxel from prodrug **16** after *β*-D-glucuronidase activation and self-immolation of the nitro-containing linker.

Monneret et al. [22] synthesized a glucuronide-based prodrug of paclitaxel for use in the ADEPT strategy. This three-component prodrug **16** was composed of a *β*-D-glucuronidase-cleavable moiety, a nitro-containing linker and the paclitaxel part (Scheme 20). Remarkably, this prodrug **16** was approximately 2000-fold more soluble in water than paclitaxel. Regarding its stability, more than 95% of the prodrug was recovered after 24 h in phosphate buffer at 37 °C. Furthermore, prodrug **16** was around 700-fold less cytotoxic than free drug paclitaxel. In the presence of *β*-D-glucuronidase, enzymatic cleavage of the glucuronyl residue occurred in the first step, followed by self-immolation of the nitro-containing spacer to release the active paclitaxel (Scheme 20). The only limitation of this prodrug is the relatively inefficient enzyme activation, as a high concentration of enzyme was required for rapid release. This was attributed to the steric hindrance of the glycosidic linkage.

**Figure 2:**
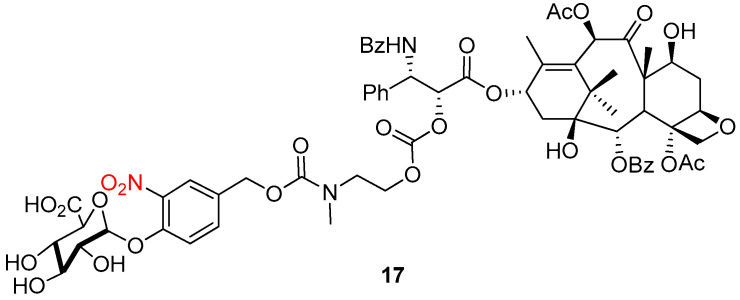
Paclitaxel prodrugs containing a carbonate moiety on the linker.

To overcome this limitation, the same group developed a prodrug **17** bearing a carbonate function that exhibits faster enzymatic cleavage than the previous carbamate moiety (Figure 2) [23].

**Scheme 21:**
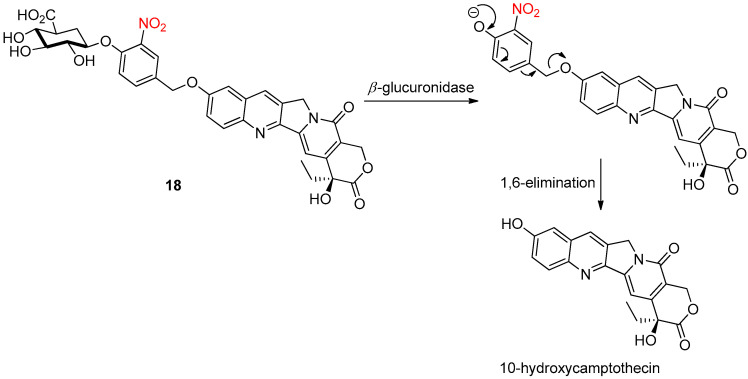
Release of 10-hydroxycamptothecin from prodrug **18** by *β*-glucuronidase activation.

Chern et al. [24] also described a glucuronide-based prodrug **18** with 10-hydroxycamptothecin, a pentacylic alkaloid with promising antitumor activity, as the liberated active compound in place of the previous paclitaxel. This prodrug **18** showed reduced cytotoxicity and improved water solubility compared with 10-hydroxycamptothecin. The 3-nitrobenzylether linker, with its strong electron-withdrawing nitro group, facilitated the enzymatic cleavage of the glycosidic bond. Then, a 1,6-elimination reaction allowed the release of active 10-hydroxycamptothecin (Scheme 21).

To optimize *β*-glucuronidase-based prodrug therapy, the use of PET imaging could be of great interest to localize and quantify the presence of *β*-glucuronidase. 

**Scheme 22:**
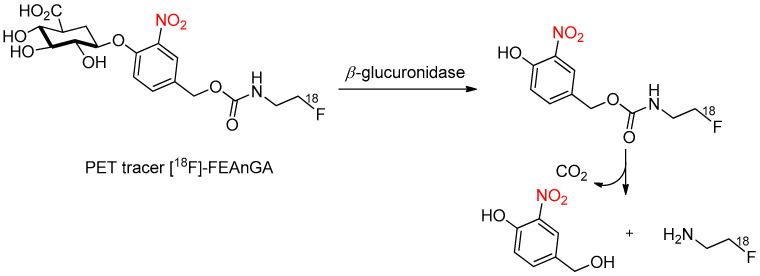
Structure of the PET tracer and its activation mechanism.

Therefore, Antunes et al. [25] synthesized a [^18^F]-FEAnGA PET tracer for extracellular *β*-glucuronidase recognition, based on a 2-[^18^F]fluoroethylamine group linked to a glucuronic acid via a self-immolative nitrophenyl linker (Scheme 22). This water-soluble prodrug was stable in aqueous buffer in the absence of *β*-glucuronidase, and the [^18^F]-FEAnGA solution remained colorless. However, upon *β*-glucuronidase activation, the prodrug was rapidly hydrolyzed and, after self-immolation of the nitrophenyl spacer, 2-[^18^F]fluoroethylamine was spontaneously released. Initial in vivo studies showed that [^18^F]-FEAnGA is a promising PET tracer for extracellular *β*-glucuronidase activity.

The ability to study the presence and localization of protein–protein complexes is of great importance for improving diagnostic capabilities. Indeed, protein–protein complexes are responsible for the diffusion of oncogenic signals, which leads to cancer development and progression. 

**Scheme 23:**
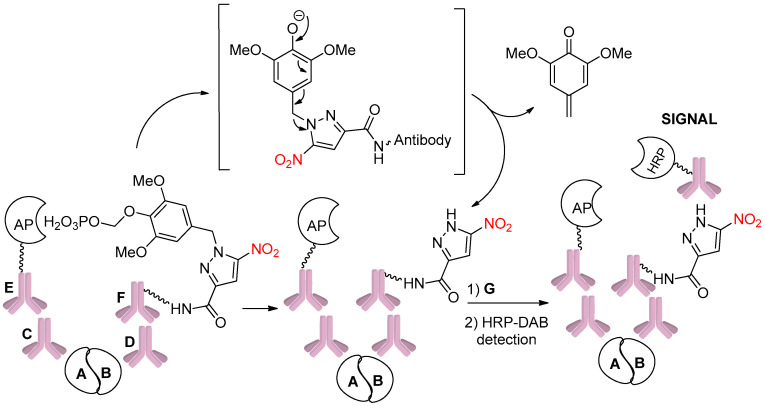
Mechanism of caged hapten protein proximity immunohistochemistry assay. (A) First protein target of interest; (B) second protein target of interest; (C) primary antibody against **A**; (D) primary antibody against **B**; (E) secondary antibody labeled with AP; (F) secondary antibody labeled with nitropyrazole-caged hapten; (G) anti-hapten tertiary antibody labeled with HRP.

Thus, Kelly et al. [26] developed a fully automated assay to detect and visualize protein–protein complexes. To this end, they modified a nitropyrazole hapten with an alkaline phosphatase (AP)-sensitive self-immolative caging group (Scheme 23). Primary antibodies **C** and **D** recognized proteins **A** and **B**, respectively, and then secondary antibody **E** labeled with AP and secondary antibody **F** labeled with a nitropyrazole-caged hapten were introduced. If there is a complex between proteins **A** and **B**, AP promotes the self-immolation of the caged hapten. Finally, the native hapten is revealed by a tertiary antibody using conventional immunohistochemistry.

**Scheme 24:**
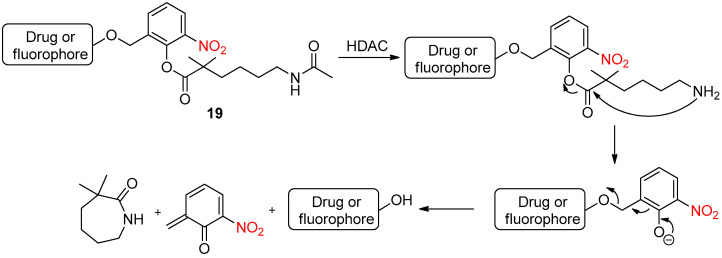
Schematic illustration of the mechanism of HDAC-mediated decaging reactions.

Histone deacetylases (HDACs) are the enzymes required for the regulation of various biological processes, specifically catalyzing the removal of the acetyl moiety. Abnormal HDAC activities are associated with several diseases, including cancers. Very recently, Jiang et al. [27] reported a reactivity-tunable self-immolative design for histone deacetylase imaging or in vivo drug delivery. The self-immolative molecule **19** was composed of three parts: an HDAC substrate, a nitro phenyl ester linker and a fluorophore for imaging applications or a drug as a leaving group (Scheme 24). 

After the deacetalyzation of compound **19** by HDAC activation, intramolecular addition of the resulting primary amine to the ester, followed by an elimination reaction, allowed the release of the drug or fluorophore. The introduction of the electron-withdrawing nitro group had two advantages: increasing the electrophilicity of the ester function and weakening the basicity of the phenolate to achieve a higher leaving ability.

## 4. NO_2_ Group on the Reporter

In this last part, after stimulation of the self-immolated compound, a nitro-containing molecule was released.

### 4.1. Chemical Activation

#### 4.1.1. Para-Nitrophenoxide Ion as a Reporter Group

As stated earlier, dendrimers are highly ordered, branched polymeric structures. Typically, dendrimers are symmetric about the core. It was demonstrated that dendrimers can disassemble via a cascade of cleavage reactions after an initial triggering event. Thus, this resulted in a high local concentration of the same dendrimer fragments, which constituted a concept called “dendritic amplification”.

**Figure 3:**
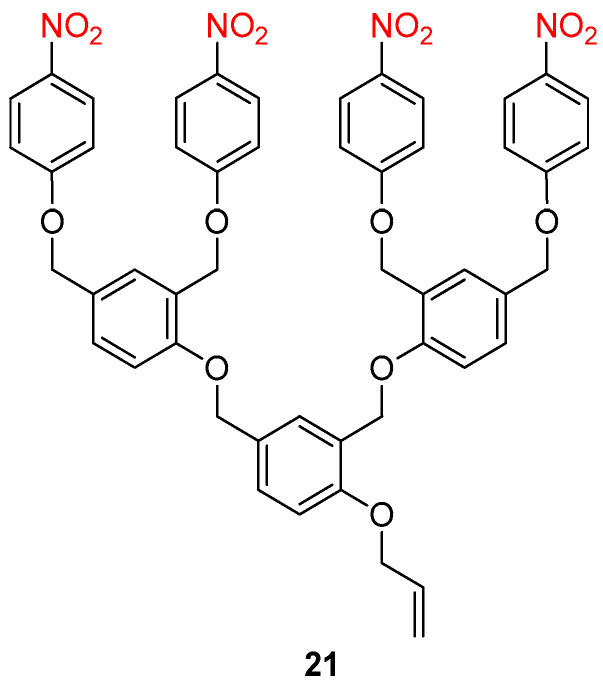
Dendrimer **21** resulting in dendritic amplification.

McGrath et al. [28] synthesized a dendritic structure **21** possessing *para*-nitrophenoxy moieties at the periphery of each dendron and an allyl trigger group (Figure 3). When deprotection of the allyl group occurred as the initial triggering event, the release of *para*-nitrophenoxide ion monitored by UV absorbance indicated complete cleavage of the dendrimer. 

**Scheme 25:**
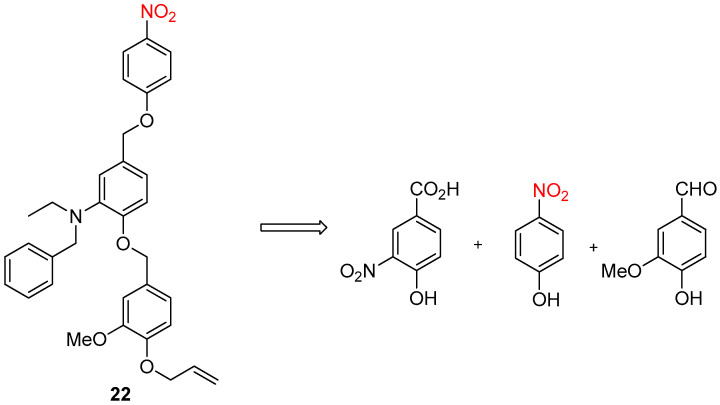
Retrosynthetic analysis of dendrimer **22** to reduce the incubation time.

Later, the same group [29] reported a significant improvement in the dendrimer’s synthesis by using 4-hydroxy-3-nitrobenzoic acid as a building block (Scheme 25). Furthermore, these modifications of the dendritic structure **22** resulted in a significantly reduced incubation period, from minutes in the previous report [30] to seconds herein.

**Scheme 26:**
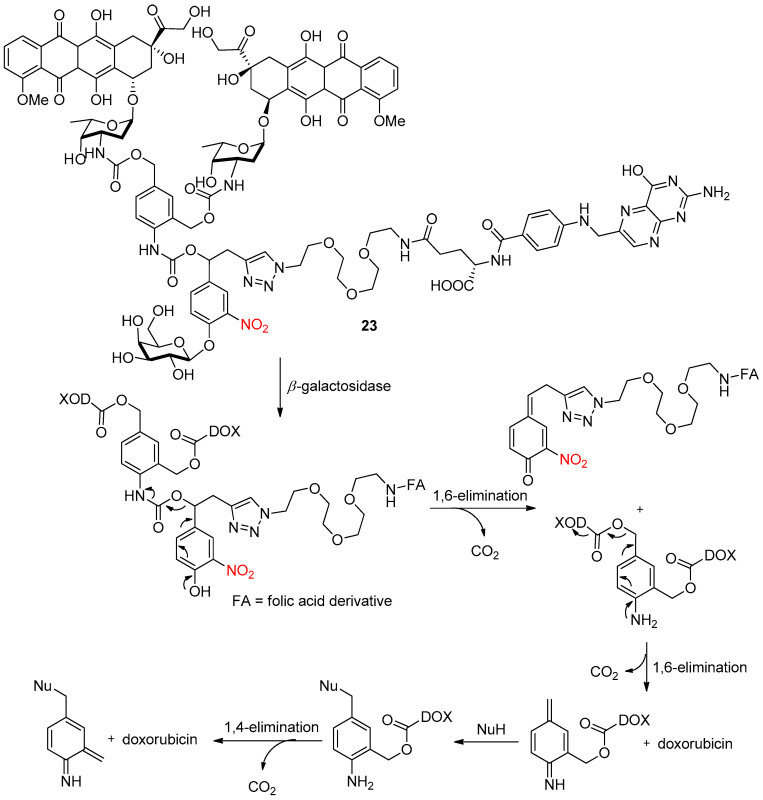
*β*-Galactosidase-catalyzed release mechanism of the two drug units of doxorubicin from the chemical system **23**.

To exploit the property of these self-immolative amplifiers, Papot et al. [31] synthesized a system **23** composed of five units, including a targeting ligand, an enzymatic trigger, a self-immolative spacer and two active drugs articulated around a chemical amplifier (Scheme 26). After *β*-galactosidase activation and subsequent multiple elimination reactions, two molecules of doxorubicin were released in tumors expressing folate receptors.

#### 4.1.2. Nitroanilines as Reporter Group

Usually, chemical activation to induce self-immolation operates via the activation or deprotection of the initial triggering group by nucleophiles. Indeed, the nucleophilic attack then initiates an electronic cascade to finally release the reporter group. 

**Scheme 27:**
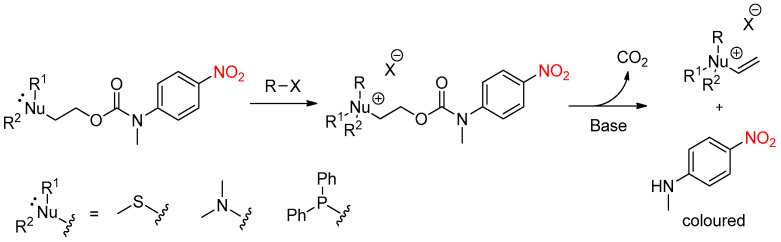
Proposed self-immolative mechanism using electrophiles.

Recently, Russell et al. [32] developed the first self-immolative systems that are triggered by reactive electrophilic alkylating agents (Scheme 27). After alkylation of the nucleophilic trigger group, the increase in the acidity of the *α*-protons allowed their deprotonation by a base, followed by self-immolative *β*-elimination with decarboxylation and the release of the colored 4-nitroaniline reporter group. 

Three possible trigger groups were studied, namely S, N and P-based derivatives. The phosphine disclosure system showed the best balance between alkylation and elimination rates. This self-immolative molecule could afford an efficient pathway for the disclosure of electrophilic species such as chemical warfare agents (nerve agents), without the need for the use of analytical instrumentation, because the nitroaniline reporter is colored.

**Figure 4:**
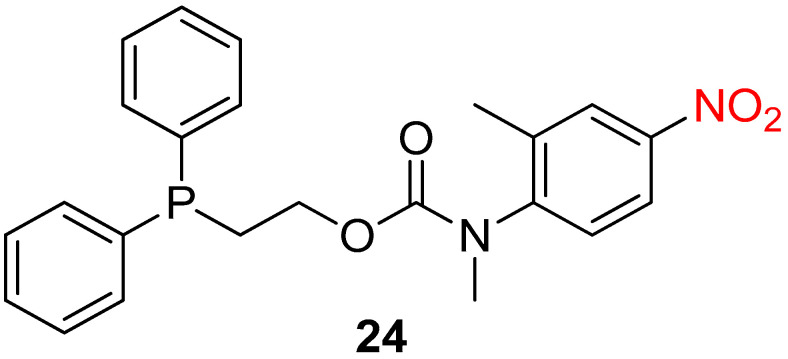
Best self-immolative system 24 for disclosure of reactive electrophilic alkylating agents.

Recently, the same group studied the influence of the reporter group on the degradation and stability of the self-immolative system [33,34]. The positions of the nitro substituent and the methyl groups on the nitroaniline unit exhibited an influence on the self-immolative efficacy of the system. Compound **24** showed an optimal balance between stability and reactivity (Figure 4).

**Scheme 28:**
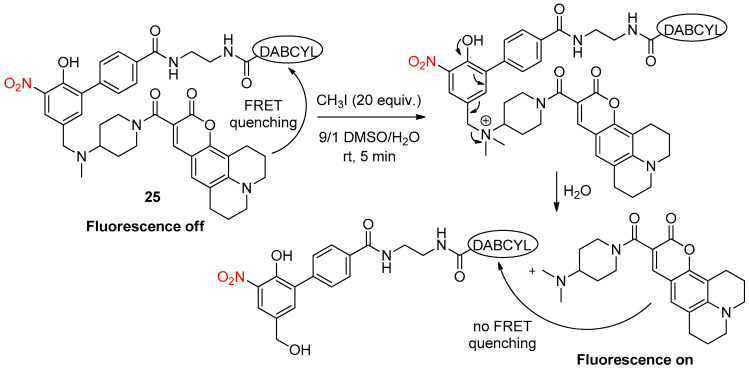
Mechanism of FRET probe **25** activation for the detection of alkylating agents.

To avoid the use of air-sensitive phosphine, Le Gall et al. [35] described a fluorescent resonance energy transfer (FRET) probe **25**, for the detection of alkylating agents, such as methyl iodide, based on a 2-nitrophenolic spacer with a nucleophilic tertiary amine at the *para* position (Scheme 28). This probe was equipped with a fluorophore (coumarin 343) and a quencher (DABCYL) that prevents fluorescence when both are still attached via the linker. However, upon the addition of the tertiary nucleophilic amine to the electrophilic alkylating agent, an intermediate tetraalkylammonium cation was formed and the self-immolation of this reactive spacer allowed the fluorophore’s release.

### 4.2. Enzymatic Activation

To better understand the physiological and pathological roles of neuraminidases, many efforts have been made to probe neuraminidases’ activity. However, most substrates are *α*-*O*-linked sialosides that, after neuraminidase-catalyzed cleavage, release hydroxyl-containing reporters for visualization. *α*-*S*-linked and *α*-*N*-linked analogs tend to be unpreferable. However, recently, a few studies showed that amine-containing reporters can be released from prodrug molecules by the enzymatic removal of the sialic acid moiety [36,37].

**Figure 5:**
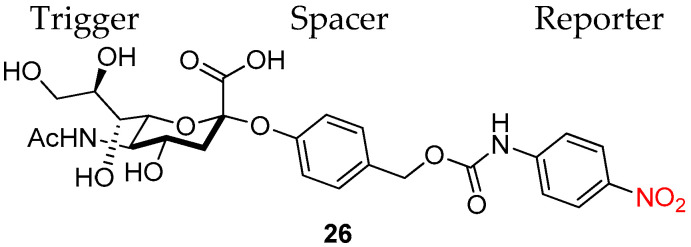
Chemical structure of the prodrug neuraminidase substrate **26**.

Inspired by these reports, Matsuoka et al. [38] developed the synthesis and enzymatic study of a neuraminidase substrate **26** containing an amine reporter. This three-component model substrate 26 was composed of a Neu5Ac trigger, a self-immolative carbamate linker and a chromogenic 4-nitroaniline reporter (Figure 5). 

**Scheme 29:**
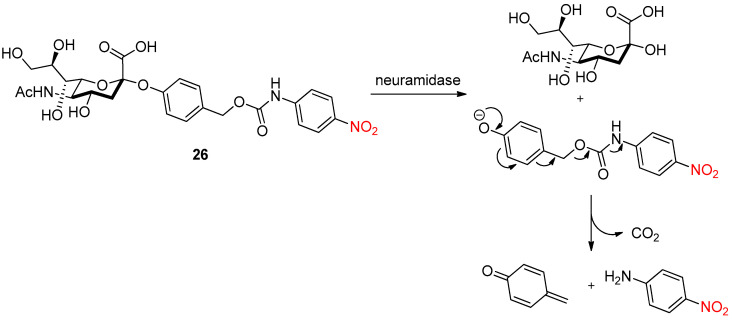
A plausible mechanism of neuraminidase-triggered activation of **26**.

After the neuraminidase-based enzymatic removal of the Neu5Ac residue, a sequential 1,6-elimination and decarboxylation reaction resulted in the release of the 4-nitroaniline reporter and *para*-quinone methide (Scheme 29).

With high concentrations of neuraminidase, the quantitative release of 4-nitroaniline was observed, and, in the absence of neuraminidase, no significant release was detected. With low concentrations, neuraminidase inactivation was observed, suggesting that the substrate likely also functioned as a neuraminidase inactivator, causing its suicide.

## 5. Conclusions

The important effect of the nitro group on the polarity of molecules has been widely used to design new self-immolative systems. Indeed, the nitro group can be reduced to the corresponding amine, whose nucleophilic character can then trigger a self-immolative cascade. In some cases, the very strong electron-withdrawing character of the nitro group has been used to improve the linker disassembly process. Finally, the release of chromogenic nitro-containing compounds as reporters has been very useful to follow the evolution of self-immolative processes. Most of these nitro-containing self-immolative molecules have found biological applications as prodrugs, but also as probes, for the detection of chemical warfare agents and in materials science. Thus, the development of new nitro-containing self-immolative molecules for biological applications is still required and relevant. 

## Data Availability

Data sharing not applicable.
